# Physiological Studies of Glutamine Synthetases I and III from *Synechococcus* sp. WH7803 Reveal Differential Regulation

**DOI:** 10.3389/fmicb.2016.00969

**Published:** 2016-06-28

**Authors:** María Agustina Domínguez-Martín, Jesús Díez, José M. García-Fernández

**Affiliations:** Departamento de Bioquímica y Biología Molecular, Campus de Excelencia Internacional Agroalimentario CEIA3, Universidad de CórdobaCórdoba, Spain

**Keywords:** *Synechococcus*, marine cyanobacteria, glutamine synthetase, physiological regulation, *glnA*, *glnN*, gene expression

## Abstract

The marine picocyanobacterium *Synechococcus* sp. WH7803 possesses two glutamine synthetases (GSs; EC 6.3.1.2), GSI encoded by *glnA* and GSIII encoded by *glnN*. This is the first work addressing the physiological regulation of both enzymes in a marine cyanobacterial strain. The increase of GS activity upon nitrogen starvation was similar to that found in other model cyanobacteria. However, an unusual response was found when cells were grown under darkness: the GS activity was unaffected, reflecting adaptation to the environment where they thrive. On the other hand, we found that GSIII did not respond to nitrogen availability, in sharp contrast with the results observed for this enzyme in other cyanobacteria thus far studied. These features suggest that GS activities in *Synechococcus* sp. WH7803 represent an intermediate step in the evolution of cyanobacteria, in a process of regulatory streamlining where GSI lost the regulation by light, while GSIII lost its responsiveness to nitrogen. This is in good agreement with the phylogeny of *Synechococcus* sp. WH7803 in the context of the marine cyanobacterial radiation.

## Introduction

A significant proportion of phytoplankton biomass production in oligotrophic seas is contributed by unicellular cyanobacteria of the genera *Synechococcus* and *Prochlorococcus* (Partensky et al., 1999; Flombaum et al., 2013; Biller et al., 2015). The majority of *Synechococcus* strains are capable of utilizing oxidized forms of nitrogen, nitrate, and nitrite (Scanlan et al., 2009), whereas the majority of *Prochlorococcus* are not (López-Lozano et al., 2002; Moore et al., 2002; Berube et al., 2015). The use of different nitrogen (N) sources by these marine picocyanobacteria is of particular interest because there is ecophysiological and genomic evidence for the critical role that nutrition plays in the ecology and evolution of these organisms (Scanlan, 2003; Flombaum et al., 2013; Biller et al., 2015; Kent et al., 2016). The spatial distribution of these two groups of marine picocyanobacteria depends on different factors such as nutrients or temperature (Partensky et al., 1999; Johnson et al., 2006; Zinser et al., 2006; Zwirglmaier et al., 2007; Pittera et al., 2014; Kent et al., 2016). *Synechococcus* spp. thrives in mesotrophic and moderately oligotrophic waters where they are able to exploit both oxidized and reduced forms of nitrogen. On the other hand, *Prochlorococcus* spp. are much more abundant in the most highly stratified, nutrient-poor waters, where they displace *Synechococcus* spp. as the dominant picocyanobacterial group.

Glutamine synthetase (GS; EC 6.3.1.2) catalyzes glutamine synthesis from glutamate and ammonia at the expenditure of ATP. Glutamine is then used by glutamate synthase (GOGAT) to synthesize glutamate by transferring an amide group to 2-oxoglutarate. The net glutamate molecule produced in each GS/GOGAT cycle is the way to incorporate nitrogen into various organic compounds such as other amino acids, purines, pyrimidines, and amino sugars. Two different types of GS have been found in cyanobacteria. Most strains have only one GS (known as GS type I, GSI), encoded by *glnA*. Prokaryotic GSI is composed of 12 identical subunits of about 50 kDa each (Valentine et al., 1968; Yamashita et al., 1989). Some cyanobacterial strains have, in addition to GSI, an hexameric GS (known as GS type III, GSIII), encoded by *glnN* (Reyes and Florencio, 1994; García-Domínguez et al., 1997; Reyes et al., 1997; Crespo et al., 1998; Sauer et al., 2000; Florencio and Reyes, 2002). Although these two types of GS are quite different in amino acid sequence, they share five domains that can be found in all known GSs (Reyes and Florencio, 1994; Crespo et al., 1998). GSIII, initially identified in an anaerobic bacterium that grows in mammalian intestines *Bacteroides fragilis* (Hill et al., 1989), is composed of six identical subunits, each of 75 kDa with little homology to GSI. This GS was the most recently discovered family among GSs and thus has been described in a limited number of bacteria including a few cyanobacterial strains, but not in eukaryotes.

The role of GSIII in cyanobacterial nitrogen metabolism is a subject that requires further investigation. Induction of GSIII under conditions of nitrogen deficiency is found in several cyanobacterial strains where GSIII coexists with GSI (Reyes and Florencio, 1994; García-Domínguez et al., 1997; Sauer et al., 2000), suggesting that the presence of GSIII may be required under this condition. The recovery rate after prolonged nitrogen starvation was negatively affected in a *glnN* mutant in *Synechococcus* PCC 7942 (Sauer et al., 2000), but the role of GSIII in that recovery remains unclear.

While nitrogen metabolism in freshwater cyanobacteria has been studied for several decades and is well known (Herrero et al., 2001; Muro-Pastor and Florencio, 2003; Flores and Herrero, 2005; Muro-Pastor et al., 2005; Luque and Forchhammer, 2008), there are very scarce studies on physiological nitrogen assimilation in marine picocyanobacteria due to their comparatively recent isolation, and their difficult culture in the laboratory. Several physiological studies in marine *Synechococcus* strains (Lindell et al., 1998, 2002; Collier et al., 1999; Lindell and Post, 2001; Bird and Wyman, 2003; Wyman and Bird, 2007; Collier and Lovindeer, 2012) and in *Prochlorococcus* (El Alaoui et al., 2001; Gómez-Baena et al., 2001, 2006; Lindell et al., 2002; López-Lozano et al., 2002, 2009; Moore et al., 2002; Kamennaya et al., 2008; Kamennaya and Post, 2011; McDonagh et al., 2012; Berube et al., 2015) have been published, but little information about the physiological behavior of GSI and GSIII from *Synechococcus* sp. WH7803 is available.

In the present work, we have studied the physiological regulation of GSI and GSIII in cultures of *Synechococcus* sp. WH7803 subjected to different ecological conditions such as nutrient limitation, different nitrogen sources or darkness. We also assessed the effect of L-methionine sulfoximine (MSX), an inhibitor of GS. We measured GS transferase activity, the concentration of both enzymes by Western blotting, and gene expression. We found that GSI from *Synechococcus* sp. WH7803 responds to nitrogen deficiency although an unusual response was found when cells were grown in darkness. Under the conditions tested in this study GSIII did not show any specific role in the assimilation of ammonium.

## Materials and Methods

### Culture Conditions

*Synechococcus* sp. WH7803 was grown in a chemically defined artificial seawater (ASW) medium (Moore et al., 2007). Cells were grown in polycarbonate Nalgene flasks (10 L), in a culture room under continuous blue light at 40 μE m^-2^ s^-1^ and 24°C. Growth was determined by measuring the absorbance of cultures at 550 nm.

### Cell Collection

Cells were collected when cultures reached *A*_550_ of 0.1–0.12 (exponential phase of growth), by centrifugation at 26,000 *g* for 8 min at 4°C using an Avanti J-25 Beckman centrifuge equipped with a JA-14 rotor. To analyze the effect of different sources of nitrogen, the pellet was washed with ASW medium without nitrogen and then resuspended in the same medium and supplemented with different nitrogen sources. For the experiments requiring darkness, culture bottles were completely covered with two layers of aluminum foil. A total of 100 μM MSX (Sigma) was dissolved in culture media. After pouring most of the supernatant and carefully pipetting out the remaining medium, the pellet for protein assays was resuspended in Tris–HCl 50 mM pH 7.5 and for RNA assays in 10 mM sodium acetate (pH 4.5), 200 mM sucrose and 5 mM EDTA. The pellet obtained was frozen at -80°C.

### Preparation of Cell Extracts

Cell extracts were broken with glass beads. After thawing, the samples were centrifuged at 16,900 *g* for 10 min and 4°C. The supernatants were poured and the pellets were resuspended in 250 μL 50 mM Tris–HCl pH 7.5. These suspensions were mixed with 140.6 mg glass beads B. Braun Melsungen AG (0.10–0.11 mm diameter). After that, five cycles of 3 min vortex–3 min ice were done. The mixtures were centrifuged at 16,900 *g* for 5 min at 4°C. Finally the supernatants were used for enzymatic activities. For western blotting some changes were introduced in the protocol in order to increase the concentration of the sample: the pellet was resuspended in 50 μL 50 mM Tris–HCl pH 7.5 and mixed with 28 mg glass beads B. Braun Melsungen AG (0.10–0.11 mm of diameter).

### GS Enzymatic Assays

Glutamine synthetase transferase activity was determined as previously described (El Alaoui et al., 2001). The composition of the reaction mixture was: 100 mM glutamine, 10 mM sodium hydroxylamine, 50 μM manganese chloride, 10 μM ADP, and 50 mM sodium arsenate in 0.2 M 3-(*N*-morpholino)propanesulfonic acid (MOPS) pH 7. One unit of enzymatic activity is the amount of enzyme that transforms 1 μmol of substrate per minute.

Protein concentration was determined using the Bio-Rad Protein Assay kit, based on the method described by Bradford (1976).

### Detection of GSI and GSIII by Western Blotting

Sodium dodecyl sulphate (SDS) electrophoresis was performed on a Mini-Protean III system (Bio-Rad) using 12% polyacrylamide resolving gels and 4% polyacrylamide stacking gels, loading 30 μg protein per lane. Proteins were transferred to a nitrocellulose membrane (Sigma) utilizing a semidry Trans-Blot SD System (Bio-Rad), for 1 h at 100 mA. After transfer the membrane was treated as follows: staining with 0.2% (w/v) Ponceau S in 5 % (v/v) acetic acid to check for equivalent protein loading and transfer efficiency; washing for 15 min with Tris-Buffered Saline and Tween 20 (TBS-T) (20 mM Tris–HCl pH 7.4, 150 mM NaCl, and 0.1% Tween 20). Blocking with TBS-T containing 1% (w/v) bovine serum albumin for 2 h and washing threefold for 15 min with TBS-T; incubation with primary antibody (anti-GSI from *Synechocystis* PCC 6803 produced in rabbit) diluted 1:5000 (v/v) in TBS-T 1% (w/v) bovine serum albumin overnight, with shaking at 4°C. Washing threefold for 15 min with TBS-T Buffer; incubation with secondary antibody (anti-immunoglobulin from rabbit produced in goat, linked with peroxidase, Sigma) diluted 1:4000 (v/v) in TBS-T for 20 min with moderate shaking at room temperature. Washing threefold for 15 min with TBS-T Buffer. Then, the immunoreacting material was detected with ECL Plus Western Blotting Detection System (Amersham), adding 1 mL solution A supplemented with 25 μL solution B. Chemiluminescent signal was detected using a LAS-3000 camera (Fujifilm) and images analyzed using Multi-Gauge V3.0 (Fujifilm).

Western blotting of GSIII was carried out following the protocol described above for GSI with these modifications: incubation with primary antibody (anti-GSIII from *Synechocystis* PCC 6803) diluted 1/4000 (v/v) in TBS-T 1% bovine serum albumin overnight, with shaking at 4°C. The incubation with secondary antibody (anti-immunoglobulin from rabbit, linked with peroxidase, Sigma) diluted 1:2000 (v/v) in TBS-T for 30 min with moderate shaking at room temperature.

### RNA Isolation

RNA was isolated from 500 mL culture samples. Cells were harvested as described above. Total RNA was extracted using TRIsure RNA Isolation Reagent (Bioline) following the manufacturer’s recommendations, with the addition of 133 μL 8 M LiCl, an additional precipitation step included at the end of the procedure to improve the RNA quality. RNA was treated with RNase-free DNaseI (Ambion) following the manufacturer’s instructions, and the absence of contaminating genomic DNA was assessed using a PCR control test.

### qRT-PCR Determination of Gene Expression

The synthesis of cDNA by reverse transcriptase (RT) from the RNA samples was carried out using the iScriptTM cDNA Synthesis kit from Quanta as recommended by the manufacturer. One microgram RNA was reverse transcribed in a 20 mL total reaction volume. Specific primers to amplify fragments of the genes from *Synechococcus* sp. WH7803 were designed using the Oligo 4.05 software (Molecular Biology Insights, Inc.), on the basis of the corresponding *Synechococcus* sp. WH7803 genome sequence (Dufresne et al., 2008). During the optimization of quantitative real-time PCR (qRT-PCR) reactions, products were checked for single amplification of DNA fragments of the expected size by agarose gel electrophoresis. The sequences of the primers used for *glnAI* were:

RT-FGS1SY: ATTTATCTGGCAGCGGTTTG

RT-RGS1SY: TTCAATGGTGTCAACGCTGT.

and for *glnN* were:

RT-FGNSY: CTCCGGAAAGCATGTGAACT

RT-RGNSY: GCAGCACAGAACAACAGGAA

Real-time quantitative PCR reactions were performed in triplicate. The reaction mixtures contained 1× concentration of SsoFast EvaGreen Supermix from Bio-Rad, 0.128–0.384 μM forward and reverse primers (depending on the efficiency calculations) and the corresponding cDNA. The efficiency of the reactions was calculated and optimized following a method described previously (Pfaffl, 2001).

An iCycler IQ multicolor real-time PCR detection system from Bio-Rad was used for quantitative detection of amplified PCR products using the following thermal cycling conditions: 95°C for 2 min, and 50 cycles of 95°C for 15 s, followed by 58°C for 30 s and 72°C for 30 s. At the end, reactions were checked to discard false amplifications by verifying the melting point of PCR products, determining the fluorescence between 65 and 100°C, with increases of 0.5°C, measured each 10 s. DNA contamination in the qRT-PCR experiments was discarded by using a no RT control sample.

The relative change in gene expression was endogenously normalized to that of the *rnpB* gene (RT-FRNSY: TGAGGAGAGTGCACAGAAA and RT-RRNSY: GTTTACCGAGCCAGCACCT), encoding RNase P, calculated using the ^2^-ΔΔC_t_^^ method (Pfaffl, 2001). No change in *rnpB* gene expression was observed under our experimental conditions.

### Genomic Sequences and Phylogenetic Analysis

All available cyanobacterial *glnA* and *glnN* sequences were retrieved from CYORF^1^, Cyanobase^2^, Integrated Microbial Genomes^3^, and NCBI^4^ databases. Molecular weight and p*I* values of the corresponding GS subunits were calculated using the tool compute pI/MW from ExPASy^5^. Alignment of deduced protein sequences and phylogenetic tree were obtained using JalView 2.9 0b2 (Waterhouse et al., 2009) and MEGA 7 (Kumar et al., 2016). Protein BLAST analysis were performed using NCBI BLASTp tool^6^.

### Statistical Analysis

Experiments were carried out with at least three independent biological samples. Results are shown with error bars corresponding to the standard deviation. Significance of data was assessed by using the Student’s *t*-test, and indicated in figures with asterisks: ^∗^*p* ≤ 0.05; ^∗∗^*p* ≤ 0.01.

## Results

### Phylogeny of GSIII from *Synechococcus*

Information about GSIII from marine and freshwater cyanobacteria is summarized in **Table 1**. All analyzed picocyanobacteria harbor the *glnA* gene (encoding type I GS). Surprisingly, searches showed that *Synechococcus* sp. RS9917 had two GSIII enzymes while no GSI was found. This is a rare case amongst cyanobacteria, but not the only one. It has been described that the cyanobacterium *Pseudanabaena* sp. PCC 6903 possesses only GSIII and is devoid of GSI (Crespo et al., 1998). As can be seen in **Table 1**, the length of the nucleotide and amino acid sequences are highly similar among the different strains, the molecular weights are also close (from 76.8 to 79.4 kDa). The estimated size of GSIII from *Synechococcus* sp. WH7803 was confirmed by Western blotting (**Figures 4B**, **7C** and **9C**). The main difference found among the various GSIII enzymes was primary amino acid sequence (**Table 1**). The highest similarity was between the GSIII from *Synechococcus* sp. WH7805 and *Synechococcus* sp. WH7803 (94% identity). The lowest similarities were obtained between sequences from the freshwater cyanobacteria with respect to *Synechococcus* sp. WH7803, around 63 and 65% identity. The size of the GSIII enzymes sequenced to date is approximately 250 amino acids longer than GSI.

**Table 1:** Summary of the characteristics of cyanobacterial GSIII enzymes.

Cyanobacterial strain	Gene name	Gene length (bp)	Protein length (aa)	Molecular weight (kDa)	Isoelectric point	% Identity with respect to GSIII from *Synechococcus* sp. WH7803
*Synechococcus* sp. WH7803	synWH7803_1458	2,172	723	79.4	5.93	–
*Synechococcus* sp. CC9311	Sync1253	2,125	724	79.7	6.07	91
*Synechococcus* sp. PCC 7002	SYNPCC7002_A0246	2,175	724	79.3	5.34	64
*Synechococcus* sp. WH7805	WH7805_04111	2,171	723	79.1	5.82	94
*Synechococcus* sp. RS9917	RS9917_13733	2,171	723	79.3	5.38	72
*Synechococcus* sp. RS9917_2	RS9917_10876	2,171	723	79.4	6.01	87
*Synechococcus* sp. WH5701	WH5701_08944	2,168	722	78.7	5.37	71
*Synechococcus* sp. WH8016	Syn8016_0892	2,172	723	79.7	6.04	91
*Synechococcus* sp. WH8020	Ga0078671_11684	2,172	723	79.7	5.93	90
*Synechococcus* sp. CB0101	SCB01_010100008472	2,169	722	78.3	5.74	83
*Synechococcus* sp. CB0205	SCB02_010100012355	2,169	722	78.7	5.95	83
*Synechococcus* sp. NKBG 042902	BG2DRAFT_00333	2,175	724	79.4	5.35	79
*Synechococcus* sp. PCC 8807	SYNPCC88007DRAFT_00002990	2,175	724	79.4	5.35	79
*Synechococcus* sp. PCC 73109	SYNPCC73109DRAFT_00003010	2,175	724	79.4	5.35	79
*Synechococcus* sp. NKBG15041	BG1DRAFT-00523	2,214	737	80.7	5.33	79
*Acaryochloris* sp. CCMEE 5410	ACCM5_010100030375	2,175	724	79.2	5.16	78
*Acaryochloris marina* MBIC11017	AM1_0336	2,175	724	79.2	5.24	78
*Synechococcus* sp. PCC 7335	57335_2917	2,184	727	78.6	5.03	77
Rubidibacter lacunae KORDI 51-2	KR51_0034460	2,175	724	79.3	5.5	77
*Synechocystis* sp. PCC 6803	sIr0288	2,175	724	79.2	5.29	63
*Synechococcus* sp. PCC 7942	SynPCC7942_0169	2,172	723	78.9	5.22	65


*The nucleotide and amino acid sequences were retrieved from different databases as explained in Section “MATERIALS AND METHODS.” The theoretical calculation of molecular weight and isoelectric point was obtained using the EVAly tool. % Identity was calculated using NCBI BLAST (http://blast.ncbi.nlm.nih.gov/Blast.cgi?PAGE=Proteins), using the GSIII sequence from *Synechococcus* sp. WH7803 as reference. The table shows all marine cyanobacterial *glnN* sequences available to date, including two freshwater strain sequences as a reference (*Synechocystis* sp. PCC 6803 and *Synechococcus* sp. PCC 7942).*

**FIGURE 1:**
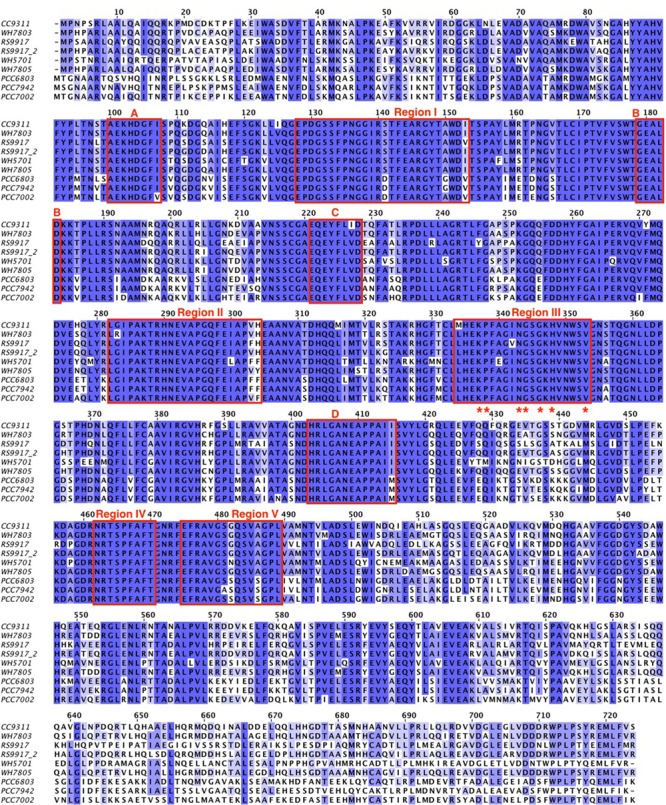
**Alignment of different GSIII from cyanobacteria.** The amino acid sequences were retrieved from different databases as explained in the Section “MATERIALS AND METHODS.” The cyanobacteria used were: WH7803 (*Synechococcus* sp. WH7803), CC9311 (*Synechococcus* sp. CC9311), PCC7002 (*Synechococcus* sp. PCC 7002), RS9917 (*Synechococcus* sp. RS9917), RS9917_2 (the second GSIII annotated from *Synechococcus* sp. RS9917), WH5701 (*Synechococcus* sp. WH5701), WH7805 (*Synechococcus* sp. WH7805), PCC6803 (*Synechocystis* sp. PCC 6803), and PCC7942 (*Synechococcus* sp. PCC 7942). The alignment was performed using the software JalView 2.9 0b2 and refined manually. The color is based on the % identity to a consensus sequence. The boxes marked with I, II, III, IV, and V are the regions typical from GSs and the boxes A, B, C, and D are the regions that defined GSIII.

An alignment of the deduced amino acid sequences of the *glnN* gene of several marine and freshwater cyanobacteria is shown in **Figure 1**, and **Supplementary Figure S1** provides an alignment of the available *glnN* sequences in cyanobacteria. The five typical regions conserved in the GS enzyme (Reyes and Florencio, 1994) are highlighted with a square. These regions correspond to five β-sheets involved in the GS active site and conserved regions (Yamashita et al., 1989; Reyes and Florencio, 1994). Region I is characterized by the sequence DGSS. Glutamic acid residues present in regions II and V are ligands to the Mn^2+^ metal associated with the active site. Region III is defined as the putative ATP binding site, seven residues are identical between the GSI, GSII, and GSIII (marked with asterisks). Finally, region IV is the glutamate-binding site and its sequence, NR-XXX-P-X-P, is conserved among the GSIII shown in **Figure 1**. The highlighted boxes represent typical conserved regions of GSIII enzymes sequenced to date (Crespo et al., 1998).

**FIGURE 2:**
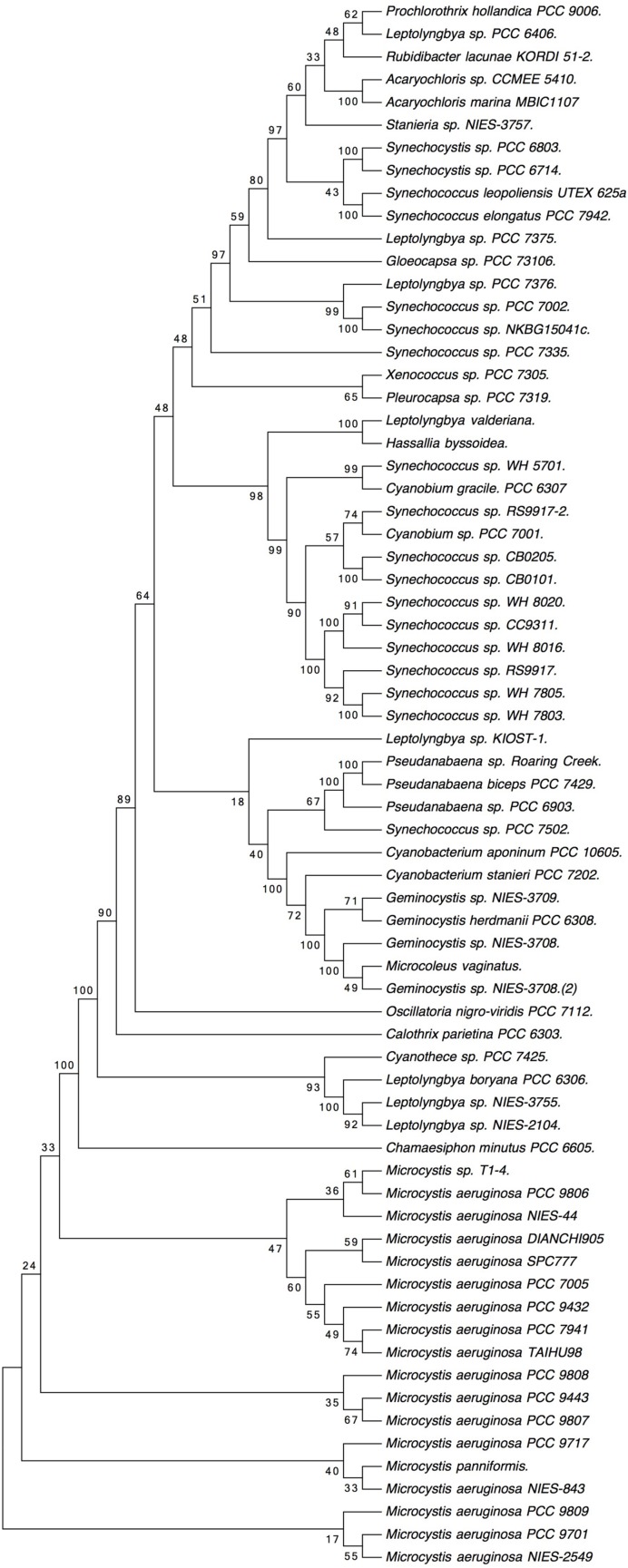
**Phylogenetic tree based on cyanobacterial GSIII sequences, obtained from the alignment shown in **Supplementary Figure S1**.** Molecular phylogenetic analysis by maximum likelihood method. The evolutionary history was inferred by using the maximum likelihood method based on the Whelan and Goldman model (Whelan and Goldman, 2001). The bootstrap consensus tree inferred from 100 replicates is taken to represent the evolutionary history of the taxa analyzed (Felsenstein, 1985). Branches corresponding to partitions reproduced in less than 50% bootstrap replicates are collapsed. The percentage of replicate trees in which the associated taxa clustered together in the bootstrap test (100 replicates) are shown next to the branches. Initial tree(s) for the heuristic search were obtained automatically by applying neighbor-join and BioNJ algorithms to a matrix of pairwise distances estimated using a Jones, Taylor and Thornton (JTT) model, and then selecting the topology with superior log likelihood value. A discrete Gamma distribution was used to model evolutionary rate differences among sites (five categories; +G, parameter = 0.7824). The rate variation model allowed for some sites to be evolutionarily invariable ([+I], 16.3612% sites). The analysis involved 69 amino acid sequences. All positions with less than 95% site coverage were eliminated. That is, fewer than 5% alignment gaps, missing data, and ambiguous bases were allowed at any position. There were a total of 722 positions in the final dataset. Evolutionary analyses were conducted in MEGA7 (Kumar et al., 2016).

The phylogenetic tree shown in **Figure 2** was deduced from the full alignment from **Supplementary Figure S1**. It grouped the sequences from marine *Synechococcus glnN* genes in a cluster which includes two sequences from *Cyanobium*. The *Microcystis* sequences are clearly separated from the rest.

*Synechococcus* sp. WH7803 possesses GSI encoded by *glnA* and GSIII encoded by the *glnN*. There are scarce physiological studies about GSI and none about the role of GSIII in marine cyanobacteria. Therefore, we set out to clarify their physiological functions.

### Nitrogen Source-Mediated Regulation

**FIGURE 3:**
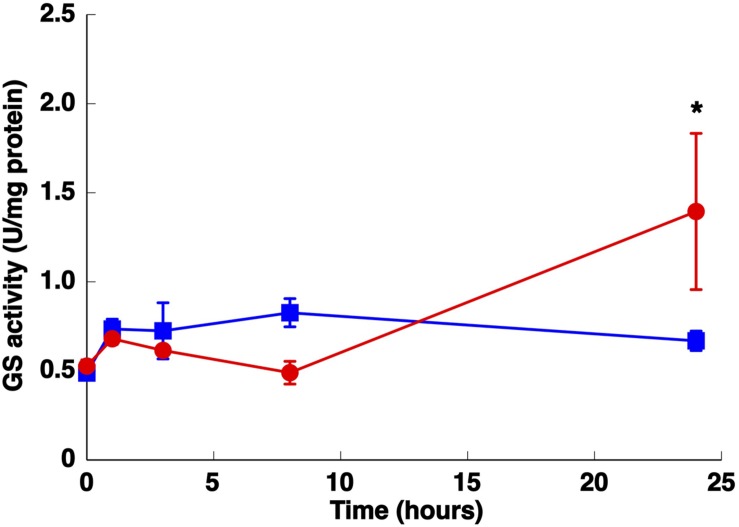
**Effect of nitrogen starvation on GS activity.** Cultures were subjected to nitrogen starvation and aliquots were taken at the indicated times. The graph represents four independent biological replicates. Error bars correspond to standard deviation. Squares, control (800 μM of ammonium); circles, nitrogen starvation. ^∗^*p* ≤ 0.05.

**FIGURE 4:**
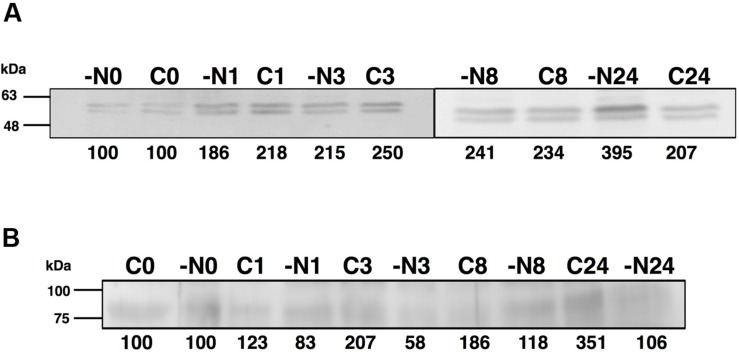
**Effect of nitrogen starvation on GSI **(A)** and GSIII **(B)** concentration.** Cultures were subjected to nitrogen starvation and aliquots were taken at the indicated times. Western blotting was performed as described in the Section “Materials and Methods.” Lanes are marked with C (control) and -N (nitrogen starvation), followed by sampling time (in hours). Quantification of bands below the picture, 100% corresponds to the intensity of the control sample at time 0.

**FIGURE 5:**
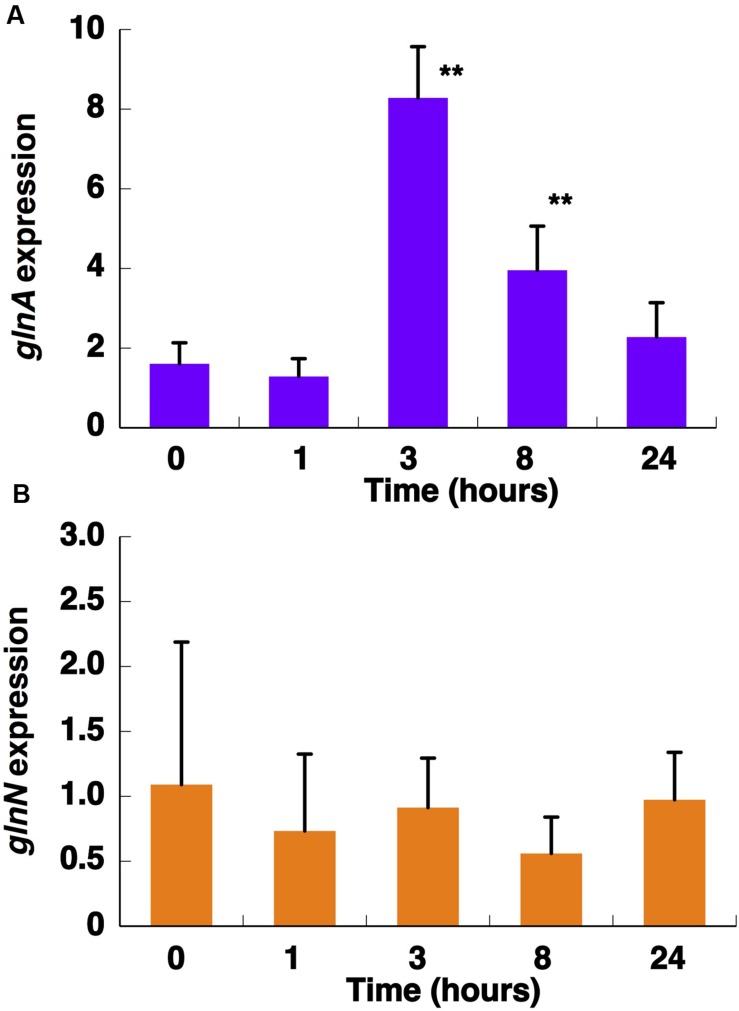
**Effect of nitrogen starvation on *glnA***(A)** and *glnN***(B)** expression.** Cultures were subjected to nitrogen starvation and aliquots were taken at the indicated times. Gene expression was measured by quantitative real-time PCR (qRT-PCR). Data are the average of four independent biological replicates. Error bars correspond to standard deviation. ^∗∗^*p* ≤ 0.01.

Previous studies have shown that several conditions, such as darkness and nitrogen starvation, cause strong effects on GS regulation in most freshwater cyanobacteria but have little effect on the *Prochlorococcus* enzyme (El Alaoui et al., 2001, 2003). Therefore, we set out to study the effect of these parameters on the activity and concentration of GS from *Synechococcus* sp. WH7803 in order to compare the results obtained with those described for *Prochlorococcus*. It is important to note that the medium used for *Synechococcus* growth was artificial, so there was no trace of nitrogen from using natural seawater. GS activity showed a significant increase (*p* = 0.0166) after 24 h under nitrogen starvation (**Figure 3**) in agreement with an increase in its concentration (**Figure 4A**). Similar results were described in *Synechococcus* sp. WH7805, where GS activity was higher after 25 h of nitrogen starvation than in the presence of ammonium (Collier and Lovindeer, 2012). Similarly, *glnA* gene expression reached a maximum peak at 3 h of nitrogen starvation (**Figure 5A**; *p* = 0.0001). These results suggest that GS from *Synechococcus* sp. WH7803 is up-regulated by nitrogen starvation, this being the standard response in most photosynthetic organisms including cyanobacteria (Mérida et al., 1991). Moreover, a potential NtcA binding site was found upstream of *glnA* in the genome of this strain. These results suggest NtcA-dependent regulation of this gene. *ntcA* expression has been shown to be negatively regulated by ammonium in *Synechococcus* sp. WH7803 (Lindell et al., 1998). Furthermore, the expression of *ntcA* in this strain was induced when cells were exposed to nitrogen stress (Lindell and Post, 2001).

The concentration of GSIII did not significantly change along the nitrogen deprivation time course, in good agreement with *glnN* expression (**Figures 4B** and **5B,** respectively). This fact differs from what is described to date in freshwater cyanobacteria such as *Synechocystis* PCC 6803 (Reyes et al., 1997) or *Synechococcus* PCC 7942 (Sauer et al., 2000): in both organisms, *glnN* was highly up-regulated after nitrogen depletion. Besides, in the upstream region of *glnN* we detected two non-canonical sites for NtcA without a corresponding TATA box (**Supplementary Figure S2**). A closer canonical NtcA binding site including a TATA box was found 500 nucleotides upstream of the putative *glnN* transcription start site. These characteristics suggest that this gene might not be regulated by this transcriptional factor in *Synechococcus* sp. WH7803. However, previous studies have shown that an imperfect NtcA binding motif upstream of *glnN* allowed up-regulation of its expression under nitrogen starvation in *Synechococcus* sp. PCC 7942 (Sauer et al., 2000).

**FIGURE 6:**
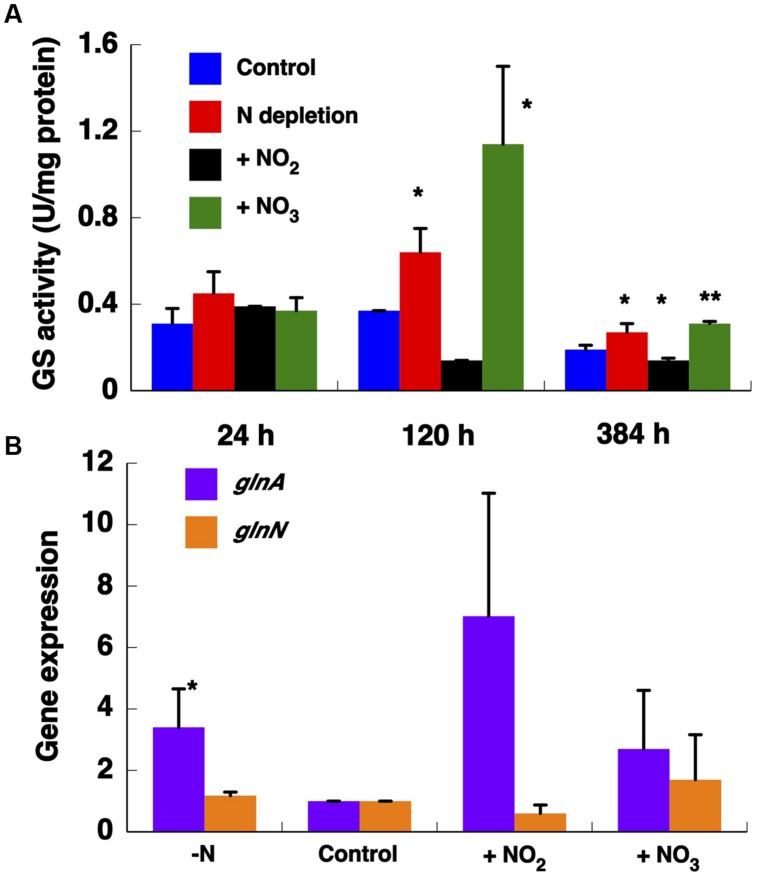
**Effect of different nitrogen sources on GS activity **(A)** and on the expression of GSs **(B)**.** The cultures were subjected to nitrogen starvation, 800 μM ammonium (control), 800 μM nitrate, and 800 μM nitrite. The cells were collected after 24, 120, and 384 h of treatment. The chart represents the average of three independent biological replicates. Error bars correspond to standard deviation. Gene expression was measured by qRT-PCR with samples after 384 h growing with different nitrogen sources. ^∗^*p* ≤ 0.05; ^∗∗^*p* ≤ 0.01.

We studied GS activity in *Synechococcus* sp. WH7803 cultures at different times after transfer to medium containing different nitrogen sources or without nitrogen; urea was not tested, as *Synechococcus* sp. WH7803 lacks the genes required for its assimilation (Collier et al., 1999). The highest GS activity was found after 120 h growth with nitrate as nitrogen source (**Figure 6A**). Longer-term experiments were performed in order to further analyze the response of GS to those nitrogen sources. After 384 h growth on the different nitrogen sources, GS activity was significantly higher under nitrogen starvation (*p* = 0.0363) and with nitrate (*p* = 0.0007) compared to the ammonium grown control. GS activity significantly decreased (*p* = 0.0179) when the cultures were grown with nitrite as nitrogen source.

The expression of both genes encoding GS was determined after 384 h (**Figure 6B**). We observed that *glnN* expression was not affected after transfer to different nitrogen sources, in contrast to the pattern found for *glnA* whose expression was significantly up-regulated under nitrogen depletion (*p* = 0.0247). These results are consistent with the observed variation in GS activity. However, the highest *glnA* expression was detected after transfer to nitrite medium (*p* = 0.0596) in contrast to the activity shown in **Figure 6A**.

### Physiological Response to Darkness

**FIGURE 7:**
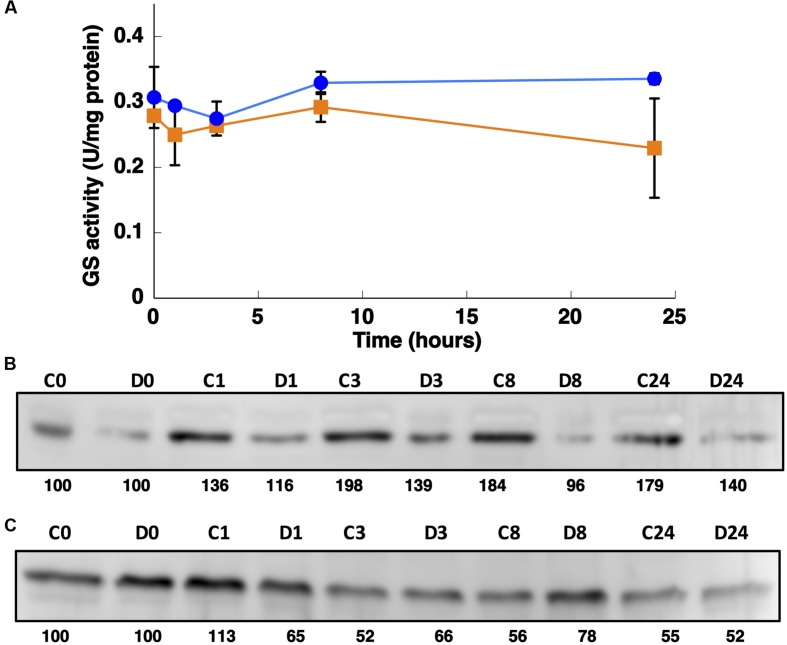
**Effect of darkness on GS activity **(A)** and the concentration of GSI **(B)** and GSIII **(C)**.** *Synechococcus* sp. WH7803 was subjected to darkness (squares) and 40 μE m^2^ s^-1^ blue light (control, circles) and aliquots were taken at the indicated times. The graph represents four independent biological replicates. Errors bars correspond to standard deviation. **(B,C)** Lanes are marked with C (control) and D (darkness), followed by sampling time (in hours). Quantification of bands below the picture, 100% corresponds to the intensity of the control sample at time 0.

The GSs of most photosynthetic organisms, including cyanobacteria, are regulated by light (Rowell et al., 1979; Marqués et al., 1992; Flores and Herrero, 1994). Hence, the effect of darkness was investigated. The growth of *Synechococcus* sp. WH7803 on ammonium-containing medium (800 μM ammonium) under darkness decreased slightly (not shown) and the GS activity remained almost unchanged even after 24 h of darkness (**Figure 7A**). This is an unusual response, as darkness promotes the down regulation of GS in most studied cyanobacteria (Rowell et al., 1979; Marqués et al., 1992; Flores and Herrero, 1994). This uncommon response was also described for *Prochlorococcus* PCC 9511 (El Alaoui et al., 2001). The concentration of the enzyme in *Synechococcus* sp. WH7803 showed a decrease after darkness, in contrast to the lack of effect of darkness on GS activity (**Figure 7B**). The same fact was described in *Prochlorococcus* SS120, a decrease of 20% of the GS concentration in crude extracts was found after 24 h darkness (El Alaoui et al., 2001).

**FIGURE 8:**
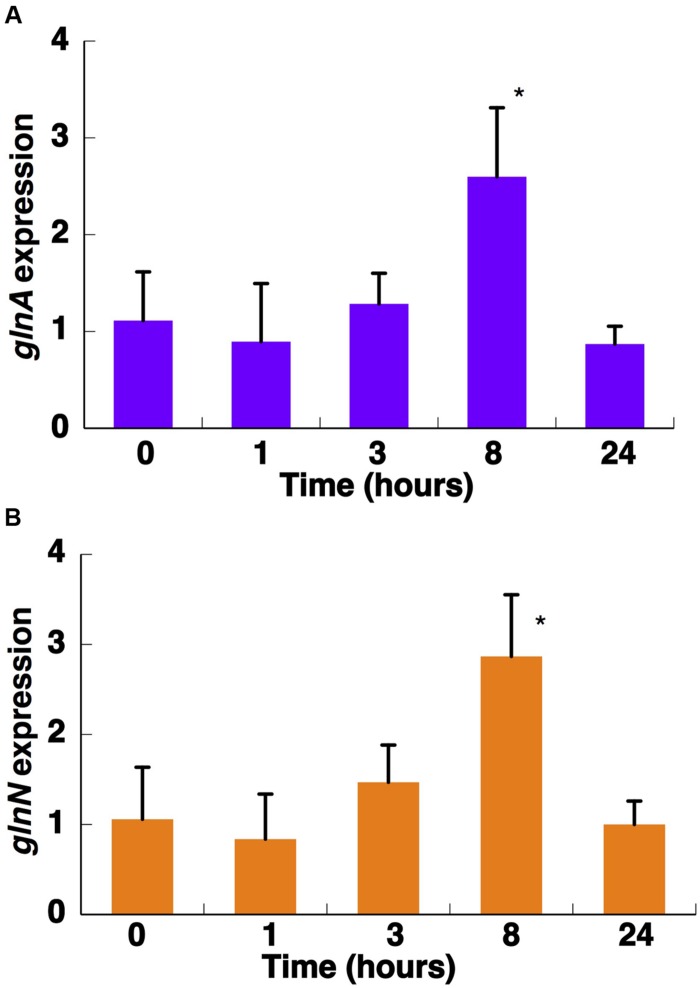
**Effect of darkness on the expression of *glnA***(A)** and *glnN***(B)**.** Cultures were subjected to darkness and 40 μE m^2^ s^-1^ blue light (control) and aliquots were taken at the indicated times. Gene expression was measured by qRT-PCR. The chart represents the average from two independent biological replicates. Error bars correspond to standard deviation. ^∗^*p* ≤ 0.05.

Interestingly, the expression of *glnA* and *glnN* showed an identical pattern under darkness (**Figure 8**). Both were, however, up-regulated significantly after 8 h darkness (*p* = 0.0419 for *glnA* and *p* = 0.0242 for *glnN*). *glnA* gene expression did not match the level of the GSI protein, in contrast to *glnN*, which was is in good agreement with the level of the GSIII protein found (**Figure 7C**), with a peak of expression and accumulation of the protein occurring after 8 h darkness (**Figure 8**). However, a clear effect on the expression of *glnA* in *Prochlorococcus* SS120, which decreased ca. seven times, has been observed after 24 h under darkness (López-Lozano et al., 2009). Besides, it has been established in other cyanobacteria that the regulation of nitrogen metabolism is affected by darkness, since the expression of the global regulator, *ntcA*, decreases under this condition in *Synechocystis* sp. PCC 6803 (Alfonso et al., 2001). Our results on GS protein concentration were consistent with this model, there was a decrease in the GSI concentration under darkness (**Figure 7B**). Nevertheless, GSIII concentration did not show a clear pattern in the absence of light.

### Effect of the GS Inhibitor MSX

**FIGURE 9:**
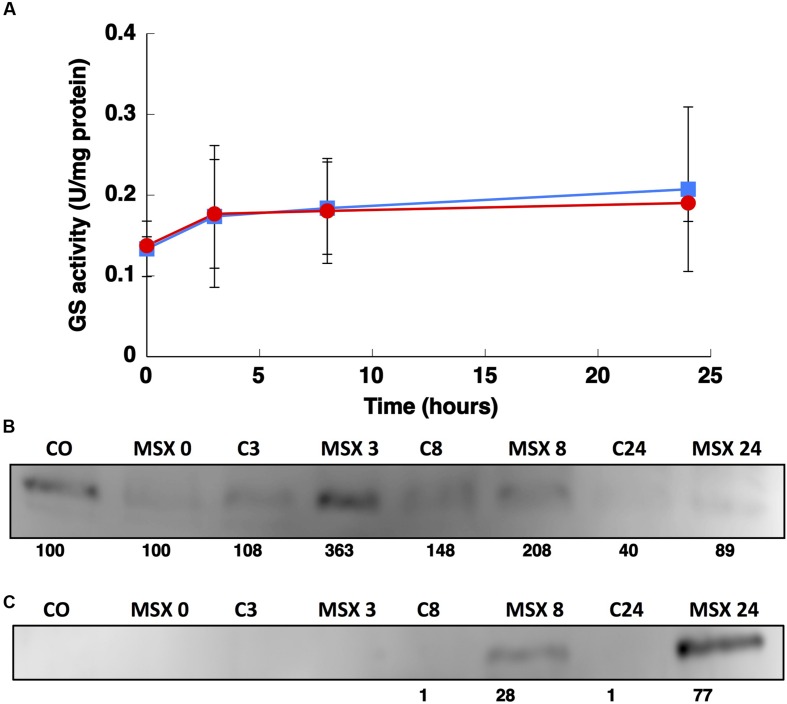
**Effect of MSX on GS activity **(A)** and the concentration of GSI **(B)** and GSIII **(C)**.** A total of 100 μM MSX was added to the culture and aliquots were taken at the indicated times. The graph represents four independent biological replicates. Error bars correspond to standard deviation. Control samples are shown with squares and MSX-treated samples with circles.

The effect of 100 μM MSX, blocking specifically the GS enzyme (Pace and McDermott, 1952), was also studied in cultures of *Synechococcus* sp. WH7803. As shown in **Figure 9A**, GS activity was not affected by the addition of 100 μM MSX. It is possible that the existence of two GSs in *Synechococcus* sp. WH7803 could compensate for the inhibition provoked by this treatment. If this hypothesis is true, it might mean that GSIII is not inactivated by MSX in *Synechococcus* sp. WH7803.

**FIGURE 10:**
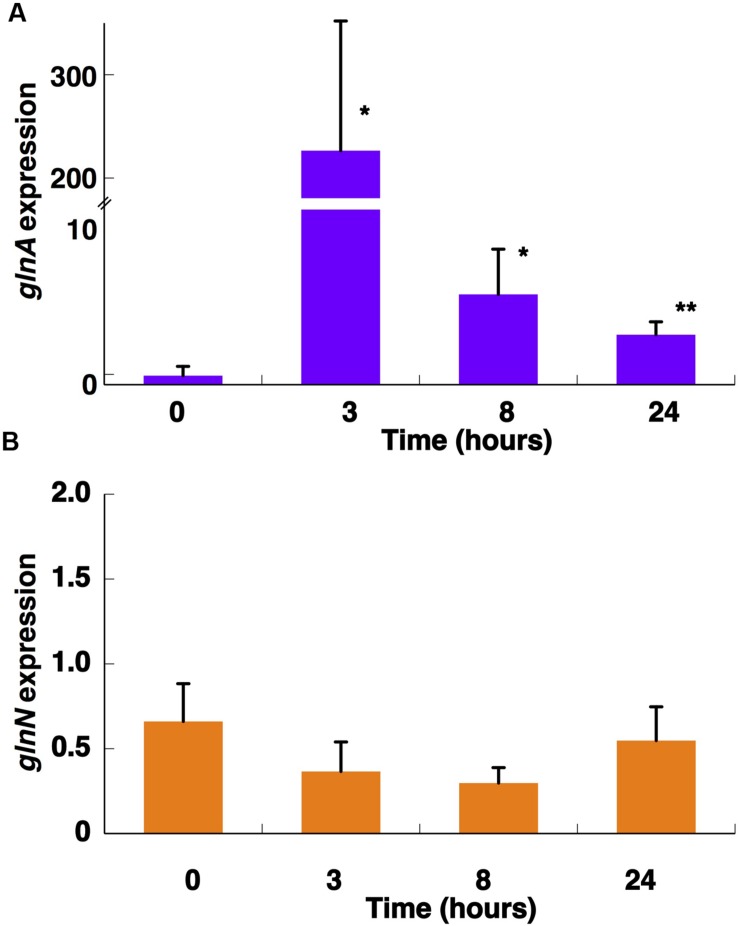
**Effect of MSX on the expression of *glnA***(A)** and *glnN***(B)**.** A total of 100 μM MSX was added to the cultures and aliquots were taken at the indicated times. Gene expression was measured by qRT-PCR. The chart represents the average from four independent biological replicates. Error bars correspond to standard deviation. ^∗^*p* ≤ 0.05; ^∗∗^*p* ≤ 0.01.

The inhibitor induced a quick rise in the expression of *glnA* (3 h; **Figure 10A**; *p* = 0.0116) in good agreement with the accumulation of the protein observed using Western blotting (**Figure 9B**). A similar response was described for *Prochlorococcus* SS120 after 1 h of the treatment, then the expression recovered to levels similar to those found at the beginning of the experiment (López-Lozano et al., 2009).

Interestingly, the expression of *glnN* was not affected by MSX treatment but the concentration of the protein increased after 8 and 24 h (**Figure 9C**). There are no other studies addressing the effect of MSX on *glnN*, so it is not possible to make direct comparisons; however, these results, together with those obtained with different nitrogen sources, suggest that *glnN* in *Synechococcus* sp. WH7803 does not respond to changes in nitrogen availability.

NtcA is a transcription factor which stimulates the expression of different nitrogen-metabolism related genes upon nitrogen stress in cyanobacteria (Luque et al., 1994). As mentioned before, *glnA* has a promoter controlled by NtcA in *Synechococcus* sp. WH7803, but *glnN* expression does not seem to be regulated by this transcription factor. Our results on gene expression (i.e., increase in *glnA* but not in *glnN* expression in nitrogen-starved cells; **Figure 5**) fit nicely with the presence of the putative NtcA-binding sites described above in *Synechococcus* sp. WH7803 (**Supplementary Figure S2**). Furthermore, our gene expression results with MSX-treated cultures (**Figure 10**) were in good agreement with previous reports addressing *ntcA* regulation in the same *Synechococcus* sp. WH7803 strain studied in this paper: *ntcA* transcript levels in ammonium-grown cells were significantly increased after MSX addition (Lindell and Post, 2001).

## Discussion

The present work is the first study analyzing in detail the changes observed in the two GSs (GSI and GSIII) from *Synechococcus* sp. WH7803 under conditions representative of the actual challenges faced by natural *Synechococcus* populations (i.e., different nitrogen sources—such as nitrate, nitrite, or ammonium—very low nitrogen concentrations in the ocean, or fluctuations in the received irradiance). Furthermore, there are no studies that show the role of GSIII in marine cyanobacteria and there are no reports on physiological regulation of GSI in WH7803. This strain is a model cyanobacterium, representative of the *Synechococcus* clades inhabiting mesotrophic areas of the ocean. Like the closely related *Prochlorococcus* it possesses a relatively streamlined genome with a small number of genes (Dufresne et al., 2008).

The *glnN* gene encodes a new type of GS that differs widely in size, structure, and amino acid sequence from previously known GSI of prokaryotes. The molecular mass of this GSIII, as deduced from the predicted amino acid sequence, is about 79.4 kDa and it was verified through Western blotting (**Figures 4B**, **7C** and **9C**). The native GSIII may be composed of six identical subunits arranged in a hexameric structure. All possible combinations of prokaryotic GSs have been shown to occur in cyanobacteria: only GSI (i.e., all *Prochlorococcus* strains thus far sequenced), only GSIII (i.e., *Synechococcus* sp. RS9917) and both GSI and GSIII (i.e., *Synechococcus* sp. WH7803; Crespo et al., 1998; Scanlan et al., 2009).

In relation to nitrogen deprivation, the results showed that GS activity from *Synechococcus* sp. WH7803 was up-regulated by nitrogen starvation (**Figure 3**), and GSI protein (**Figure 4A**) and *glnA* expression (**Figure 5A**) were higher under nitrogen deficiency. This fact was the standard response found in most photosynthetic organisms including cyanobacteria (Mérida et al., 1991; Marqués et al., 1992). However, it differs from what was found in its main competitor *Prochlorococcus* (El Alaoui et al., 2001). There is an enormous diversity in the *Prochlorococcus* radiation (Urbach and Chisholm, 1998; Rocap et al., 1999, 2002; Garczarek et al., 2007; Partensky and Garczarek, 2010), therefore the comparison between one strain of *Synechococcus*, WH7803, and the whole group of its main competitor *Prochlorococcus* is complicated. This complexity is even higher considering that *Prochlorococcus* strains only have GSI. While *Synechococcus* sp. WH7803 showed an increment in GS activity under nitrogen deprivation (**Figure 3**), it slightly decreased in *Prochlorococcus* PCC 9511 (El Alaoui et al., 2001). On the other hand, the concentration of GSI enzyme from *Synechococcus* (**Figure 4A**) increased ca. twofold compared to control samples after 24 h of nitrogen starvation, but there were only minor changes in *Prochlorococcus* (El Alaoui et al., 2001). Regarding gene expression, the increase in *glnA* expression in *Synechococcus* sp. WH7803 (**Figure 5A**) was a similar response to that found in *Synechococcus* sp. WH8103 after 8 h nitrogen deprivation (Bird and Wyman, 2003). Interestingly, a similar pattern for *Prochlorococcus* sp. SS120 (López-Lozano et al., 2009) and for MED4 and MIT9313 (Tolonen et al., 2006) was described.

The first striking result of our studies was that GSIII protein levels and *glnN* expression were not altered when the cells were transferred to medium without any nitrogen source (**Figures 4B** and **5B**). This result is in sharp contrast to those found in *Synechocystis* sp. PCC 6803 (Reyes and Florencio, 1994; García-Domínguez et al., 1997). In that case the induction of GSIII occurred only under conditions of nitrogen deficiency. Another important feature of GSIII in *Synechococcus* sp. WH7803 is that it contains a non-canonical binding site for NtcA, suggesting that it might not be regulated in a NtcA-dependent manner. This is in good agreement with the lack of response under nitrogen starvation, leading us to think that GSIII is not essential for nitrogen stress response in this strain.

*Synechococcus* usually coexists with *Prochlorococcus* in very oligotrophic regions (Partensky et al., 1999). Their competition for scarce nutrients has led to different survival strategies, including streamlining of regulatory mechanisms and differential utilization of available nitrogen sources (López-Lozano et al., 2002; Moore et al., 2002; Rocap et al., 2003; García-Fernández et al., 2004; Tolonen et al., 2006; Dufresne et al., 2008; Scanlan et al., 2009; Berube et al., 2015). This is an important factor, given nitrogen is a key element limiting growth in these regions of the ocean (Graziano et al., 1996). Most known marine *Synechococcus* strains are able to utilize oxidized forms of nitrogen. This fact represents a key difference with most *Prochlorococcus* strains isolated thus far (López-Lozano et al., 2002; Moore et al., 2002; Berube et al., 2015), although there are several strains that have been recently shown to grow on nitrate (Berube et al., 2015). With this in mind, we wanted to know how the GSs from *Synechococcus* sp. WH7803 physiologically respond to the addition of different nitrogen sources. GS activity was determined following growth with different nitrogen sources for various times. Activity was ca. threefold higher with nitrate than in control samples growing on ammonium after 120 h. Collier and Lovindeer (2012) studied GS activity in several marine *Synechococcus* strains. In all cases, the highest GS activity was observed in cultures using nitrate as nitrogen source after 25 h. These results need to be put in the context of this strain of *Synechococcus* being representative of a clade (clade V) widespread but in low abundance in various oceanic waters (Scanlan et al., 2009). Therefore, the ecological distribution of this clade extends where nitrate is available (Scanlan et al., 2009), consistent with the high GS activity in this condition.

The GSs of most photosynthetic organisms, including cyanobacteria, are regulated by light (Rowell et al., 1979; Marqués et al., 1992; Flores and Herrero, 1994). Our study found an unusual response in *Synechococcus* sp. WH7803, also described for *Prochlorococcus* PCC 9511 (El Alaoui et al., 2001), that GS activity was not affected by darkness. This is unusual since darkness promotes the down-regulation of GS activity in most studied cyanobacteria (Rowell et al., 1979; Marqués et al., 1992; Flores and Herrero, 1994). The situation found was not completely shared with *Prochlorococcus*. GS activity (**Figure 7A**) did not change under darkness, although degradation of the GSI enzyme occurred. *glnA* and *glnN* showed a peak of gene expression after 8 h darkness, the contrary pattern for *glnA* expression being found in *Prochlorococcus* sp. SS120 (López-Lozano et al., 2009). Moreover, this is the only condition in which we could observe a clear response in *glnN* expression.

The effect of MSX on GS regulation has been studied in many unicellular organisms such as cyanobacteria (Orr and Haselkorn, 1982; El Alaoui et al., 2001, 2003; López-Lozano et al., 2009; Rangel et al., 2009) and green algae (García-Fernández et al., 1995). This inhibitor provokes a rapid GS inactivation in *Anabaena* PCC 7120 (Orr and Haselkorn, 1982) and in *Prochlorococcus* sp. PCC 9511 (El Alaoui et al., 2001). However, we observed clearly different results in *Synechococcus* sp. WH7803: GS activity did not change with the addition of 100 μM MSX, although *glnA* expression was considerably higher 3 h after addition, whilst *glnN* expression did not change. Regarding the concentration of GSI in *Prochlorococcus* following MSX treatment, we found different responses depending on the strain: for SS120 the concentration of GS decreased 32%, although in the case of PCC 9511 increased 17% (El Alaoui et al., 2001).

The phylogenetic analysis of *glnA* from different marine and freshwater cyanobacteria showed that marine *Synechococcus* and *Prochlorococcus* sequences are grouped together, separated from freshwater model strains, in agreement with previous phylogenetic studies on *glnA* in marine cyanobacteria (El Alaoui et al., 2003). Combining these data with the results discussed above it can be suggested that the structure of GS has not been significantly modified in the marine picocyanobacterial clade during evolution, in contrast with the possible modulation of its regulatory properties, as previously proposed (El Alaoui et al., 2003).

This study shows that GSIII in *Synechococcus* sp. WH7803 does not have the same physiological role described for other cyanobacteria, since it is not responsive to nitrogen deficiency, unlike GSIII from *Synechocystis* sp. PCC 6803 (Reyes et al., 1997; Sauer et al., 2000), *Pseudanabaena* sp. PCC 6903 (Crespo et al., 1998) and *Synechococcus* sp. PCC 7942 (Sauer et al., 2000). The lack of response under different key conditions suggests that in this strain GSIII coexists with GSI but has no clear, specific functionality. Our results indicate its expression is constitutive but without any important role in the conditions tested in this study. The proposed role for GSIII in the recovery after prolonged nitrogen starvation (Sauer et al., 2000) seems rather unlikely in *Synechococcus* sp. WH7803, given its lack of responsiveness to nitrogen starvation.

Therefore, this could represent an intermediate situation in the evolution of the marine cyanobacterial radiation, since there are other marine *Synechococcus* that have lost this gene (i.e., *Synechococcus* sp. WH8102, WH8103, BL107) and all the sequenced *Prochlorococcus* lack GSIII. *glnN* from *Synechococcus* sp. WH7803 might be in the process of being lost, and the removal of its regulation by nitrogen availability could be the first sign of this process. The loss of *glnN* is understandable in a group of organisms characterized by a trend to compact their genomes (Strehl et al., 1999), and it might be similar to the process leading to the disappearance of other important genes (Kettler et al., 2007), e.g., *narB*, in recently evolved clades of marine *Synechococcus* and *Prochlorococcus* (Scanlan et al., 2009; Berube et al., 2015). Hence, this enzyme is probably not necessary in a relatively stable environment (García-Fernández et al., 2004), where the presence of a single gene encoding GS (*glnA*) might be enough for ammonium assimilation.

In this context, the *glnA*/GSI response in *Synechococcus* sp. WH7803 also supports an intermediate position in the progressive modification of its regulation during the evolution of cyanobacteria. In this way, GS regulation observed in freshwater strains such as *Synechocystis* sp. PCC 6803 (responding to both nitrogen availability and darkness; Marqués et al., 1992) would lose first the responsiveness to light, leading to the appearance of strains like *Synechococcus* sp. WH7803; the capability to respond to nitrogen starvation would be reduced in further evolved cyanobacteria, such as *Prochlorococcus* (El Alaoui et al., 2001, 2003; Lindell et al., 2002; Tolonen et al., 2006). This hypothesis is in good agreement with the intermediate phylogenetic position of *Synechococcus* sp. WH7803 in the cyanobacterial radiation (Scanlan et al., 2009).

## Conclusion

We suggest that *Synechococcus* sp. WH7803 shows regulatory features which fit in the context of a progressive streamlining, from early branching members of the cyanobacterial tree (enabled to react to changing environments) to late branching marine strains which are progressively adapted to more stable ocean niches, therefore losing the capability to perform fine regulation with respect to light and nutrient availability.

## Author Contributions

MD-M and JD performed research; MD-M, JD, and JG-F designed research, analyzed data, wrote and approved the final version of the manuscript.

## Conflict of Interest Statement

The authors declare that the research was conducted in the absence of any commercial or financial relationships that could be construed as a potential conflict of interest.

## Footnotes

1 http://cyano.genome.jp/

2 http://genome.microbedb.jp/cyanobase/

3 https://img.jgi.doe.gov

4 http://www.ncbi.nlm.nih.gov/

5 http://web.expasy.org/compute_pi/

6 http://blast.ncbi.nlm.nih.gov/Blast.cgi?PAGE=Proteins

## Supplementary Material

**FIGURE S1 | Alignment of available cyanobacterial GSIII sequences.** The amino acid sequences were retrieved from different databases as explained in the “MATERIALS AND METHODS”. Sequences were aligned with the *MEGA7* software (Kumar et al., 2016), by using the MUSCLE method with default settings.

**FIGURE S2 | Non-canonical NtcA-binding sites and putative TATA box in the region upstream glnN in the genome of *Synechococcus* sp. strain WH7803.**
