# Supplementary figures and images for: Physiological Studies of Glutamine Synthetases I and III from Synechococcus sp. WH7803 Reveal Differential Regulation
Source: Front Microbiol. 2016 Jun 28;7:969. doi: 10.3389/fmicb.2016.00969 (PMC4923085; doi:10.3389/fmicb.2016.00969)

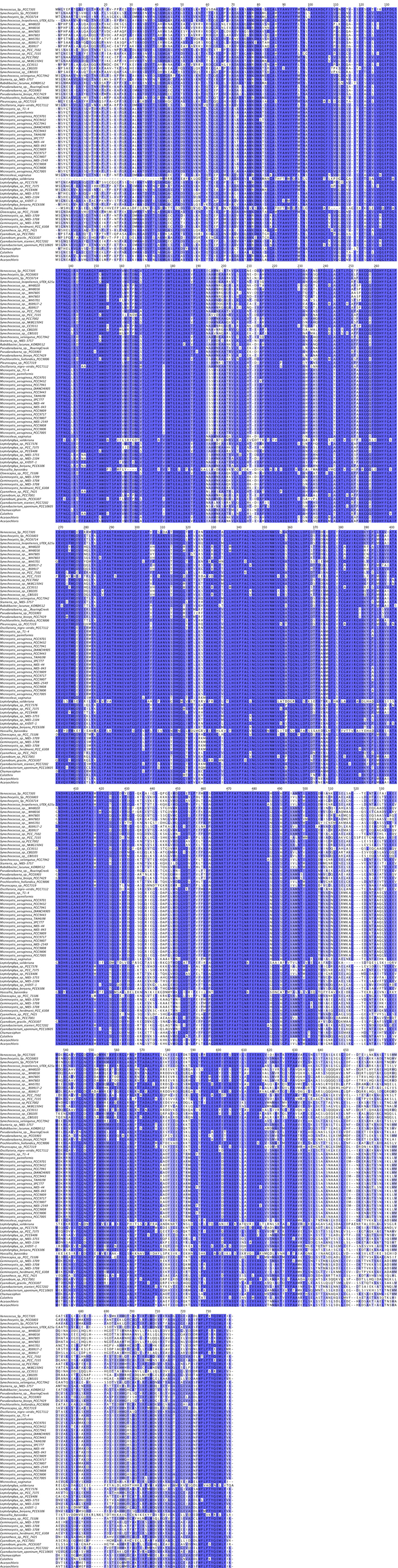

Supplement: Supplementary file 1 [file Image_1.JPEG]

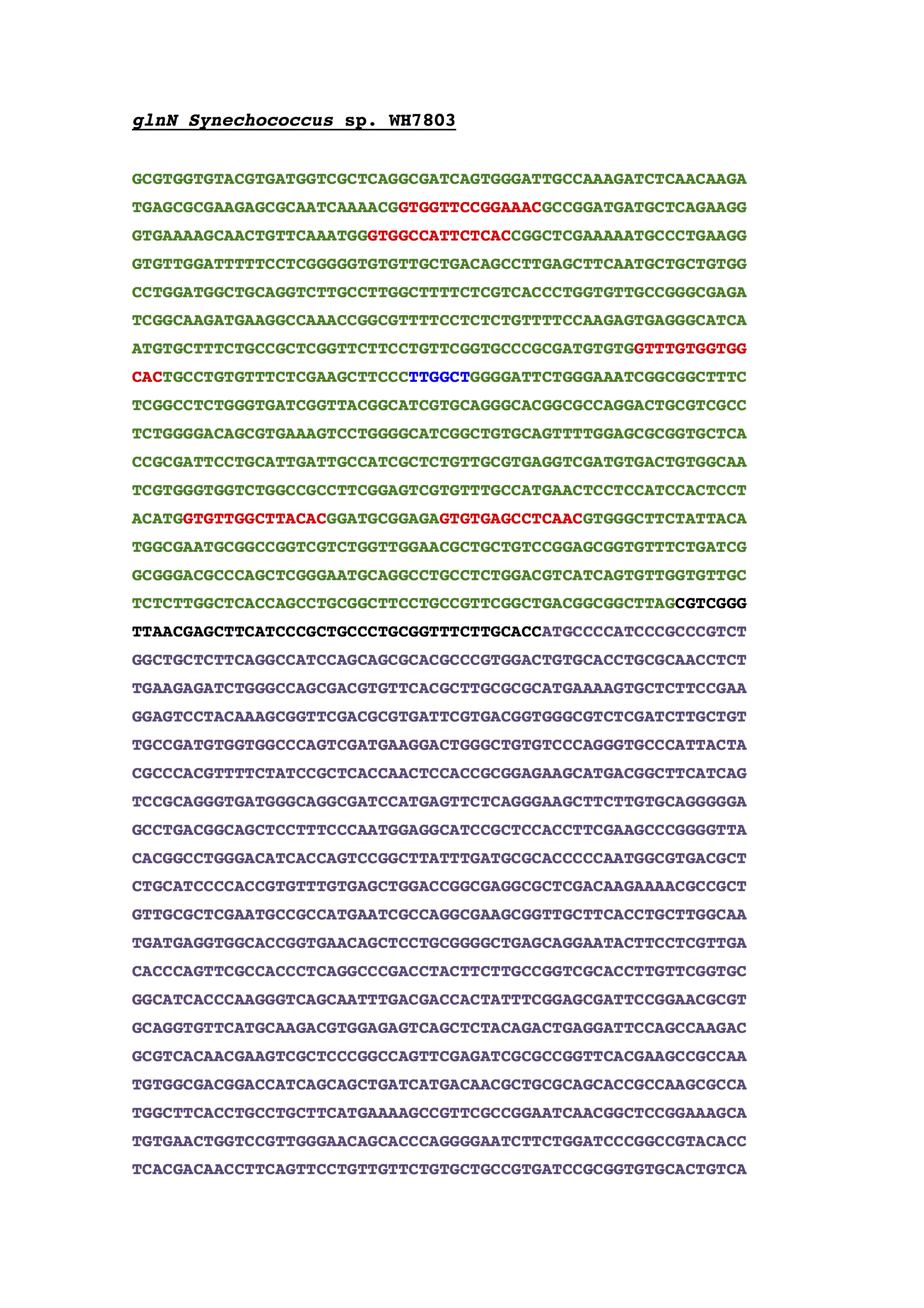

Supplement: Supplementary file 2 [file Image_2.JPEG]
